# Virginia State Veterinary Medical Association

**Published:** 1896-03

**Authors:** George C. Faville

**Affiliations:** Secretary


### VIRGINIA STATE VETERINARY MEDICAL ASSOCIATION.

THE fifth regular meeting was held in Richmond, January 2, 1896, in the Veterinary Hospital of Dr. W. H. Harbaugh. The meeting was called to order at ten o'clock A.M., by the President, Dr. W. H. Harbaugh. After the reading and approval of the minutes of the previous meeting, the report of the Board of Censors was read. Dr. Thomas M. Sweeney, V.S., of Richmond, and Dr. W. F. Henderson, M.D., of Blacksburg, were elected to membership. Dr. George Ben Johnston, M.D., of Richmond, was elected an honorary member.

The Committee on Jurisprudence and Legislation, through their chairman, Dr. W. H. Harbaugh, reported that on September 25, 1895, a negro blacksmith was tried in the police court of the city of Richmond, on the charge of cruelty to an animal, for having burnt a horse's mouth with a heated iron to cure the lampas. The case was prosecuted by the Society for the Prevention of Cruelty to Animals, and was stubbornly contested by the defense. Five veterinary practitioners, three medical doctors, one dentist, and two horsemen testified that the practice of burning for the condition called lampas was cruel and unnecessary, and many other witnesses were present to testify to the same effect, but the justice said the prosecution had introduced enough evidence. The owner of the horse testified that the horse improved after the burning. Three liverymen testified that the operation was beneficial, and that after horses were burnt they always regained their appetites. The defendant testified that he had burned between 1500 and 2000, and had never seen any bad results. Counsel for the prosecution requested that if a fine was imposed on the defendant, the fine be remitted, as this was only intended as a test-case. In summing up the justice said he must be governed by the expert evidence, and was compelled to decide that the operation was cruel, but as it had been a long-established custom, and as he did not think the blacksmith intended any cruelty, he would discharge him, but any other cases brought before him would be tried on their merits. As this was the first case of the kind ever prosecuted in this State, the Committee consider the decision of the court fair and just. They also consider it a great victory for the veterinary profession, as it will effectually check the barbarous practices of the many pretenders who dupe ignorant horse-owners. The Committee reported this case to the President of the S. P. C. A., with the request that a test-case be made of it, with the hope that a favorable decision could be obtained, in order to stop a severe infliction of pain for an entirely imaginary condition. Ever since he had been a veterinarian, Dr. Harbaugh stated, he had carefully looked for disease of the bars of young horses' mouths, and had never seen in the condition called lampas the slightest evidence of disease. No such disease exists; the condition is normal, and the ignorant should be taught not to interfere with it. Abnormal condition of the gums that requires attention does not necessitate any interference with the bars. We should cease to use the term lampas even in connection with disease of the gums.

From this same Committee a report was made upon the subject of legislation, which elicited a great amount of discussion. The action of all the States to the north of Virginia in the matter of stringent veterinary legislation, by driving a horde of quacks into this State, forces upon this Association the careful consideration of this question. It was finally agreed to ask the Legislature now in session to give us a law creating an examining board; a law practically the same as that governing the practice of medicine in this State, modified to meet the requirements of the veterinary profession.

A recess was taken to attend a clinic at the Old Dominion Hospital, by invitation of Dr. George Ben Johnston. The Association was delightfully entertained by the Doctor in the hospital amphitheatre by an entertaining talk upon the various intricacies of septic and antiseptic surgery, illustrating his remarks. Afterward the Association was entertained as the guests of Dr. Johnston, in the faculty room of the Medical College of Virginia, at an elegant collation.

The Association reconvened at 5.30 P.M., and the Committee on Resolutions (W. T. Gilchrist, chairman) reported as follows: Whereas, murrain, or Texas cattle-fever, exists in this State annually to a much greater extent than is generally known, destroying many cattle during certain seasons of the year, and almost threatening ruin to the cattle-industry of the State; and, whereas, tuberculosis exists to an alarming extent among dairy herds of the State, as proven by the best-known means of diagnosis, viz., the tuberculin test; as an instance, one herd of one hundred and thirty-four head of cattle in the vicinity of Richmond, formerly supposed to be healthy, tested by experts from the U. S. Department of Agriculture, showing 71 per cent., or ninety-five head diseased; and, whereas, Virginia has no efficient laws for the control of these or any other infectious diseases of animals; and, whereas, the U. S. Department of Agriculture has no power to interfere with the traffic of the live-stock of the State, except in so far as the Interstate Commerce Law is concerned; and, whereas, it seems to be the tendency on the part of certain governmental and other officials to suppress facts relating to the above-mentioned disease; and, whereas, the stock-breeders of Southwestern Virginia have met and appointed a committee to secure adequate laws for the control of these and other infectious diseases of animals; and, whereas, said stock-owners have recognized the fact that the veterinary profession of *this* State is the proper source from which to obtain reliable information in regard to animal diseases in this State, and have therefore appointed one of our members as a member of their committee, and, furthermore, said committee asks for the assistance of our Association; therefore, be it resolved: 1st. That we fully realize the necessity of proper laws to control and prevent the spread of contagious diseases, and that we deplore the secrecy practised in such matters at the expense of public funds and to the detriment of the true interest of the live-stock industry; 2d. That this Association aid the committee of stock-breeders from Southwestern Virginia in any way within its power so to do; 3d. That a copy of these resolutions be sent to said committee of stock-breeders, and that these resolutions be spread upon the minutes. After considerable discussion the resolutions were adopted.

The time having arrived for the reading and discussion of papers, Dr. Faville, of Norfolk, read a paper on "Osteoporosis,"[1] and was followed by Dr. M. D. Hoge, Jr., M.D., of Richmond, with a paper on "Bone-softening." The discussion following the reading of these papers was of considerable interest to the members. Dr. Hoge's paper was beautifully illustrated with microscopic specimens of affected bone, and the complete collection of specimens of osteoporosis and actinomycosis shown by Dr. Harbaugh filled the papers well. Dr. Harbaugh read a paper on "Texas Fever," which was listened to with great interest. The specific micro-organism was nicely shown, as were also the ticks found upon the cattle affected. The discussion of this disease was of peculiar interest to the members, because of the location of the government quarantine-line across the State. The results of a series of experiments and observations to be made by Drs. Harbaugh and Niles during the next season will be awaited with interest.

At 9 P.M. the Association adjourned to meet around the banquet table at Evenson's as the guest of Dr. Harbaugh. The hospitality of our President was greatly enjoyed.

At 10.30 the Association was called to order again to listen to a paper by Prof. E. P. Niles, of Blacksburg, Va., upon "Immunity."[2] This was followed by a general discussion of the subject of "Serum-therapy."

After extending the thanks of the Association to Dr. George Ben Johnston for his very profitable and pleasant entertainment, and instructing the Secretary to compile the proceedings and papers of the various Association meetings for publication, the Association adjourned at 2 A.M., January 3, to meet in Norfolk in June.

The Association has grown from a weakling of two years ago to a healthy organization, and all the members realize that the mutual benefit derived from our meetings is without measure. While in Virginia at the present time there is no legal veterinary profession, we hope and know that the influence emanating from our Association will ultimately secure for us the recognition that is our due.

GEORGE C. FAVILLE, D.V.M.,
Secretary.

### MAINE VETERINARY MEDICAL ASSOCIATION.

[1] Will appear in April number. [2] Published in this number,

